# Supplementary material for: Transient Confinement of the Quaternary Tetramethylammonium Tetrafluoroborate Salt in Nylon 6,6 Fibres: Structural Developments for High Performance Properties
Source: Materials (Basel). 2021 May 29;14(11):2938. doi: 10.3390/ma14112938 (PMC8198042; doi:10.3390/ma14112938)
Supplement: Supplementary file 1 [file materials-14-02938-s001.zip › materials-1215537-supplementary.pdf]

materials

MDPI

Supplementary Materials

Transient Confinement of the Quaternary Tetramethylammonium Tetrafluoroborate Salt in Nylon 6,6 Fibres: Structural Developments for High Performance Properties

Ahmed Dawelbeit and Muhuo Yu *

State Key Laboratory for Modification of Chemical Fibers and Polymer Materials, College of Materials Science and Engineering, Donghua University, Shanghai 201620, China; 413004@mail.dhu.edu.cn
* Correspondence: yumuhuo@dhu.edu.cn

1. Supplemental Figures

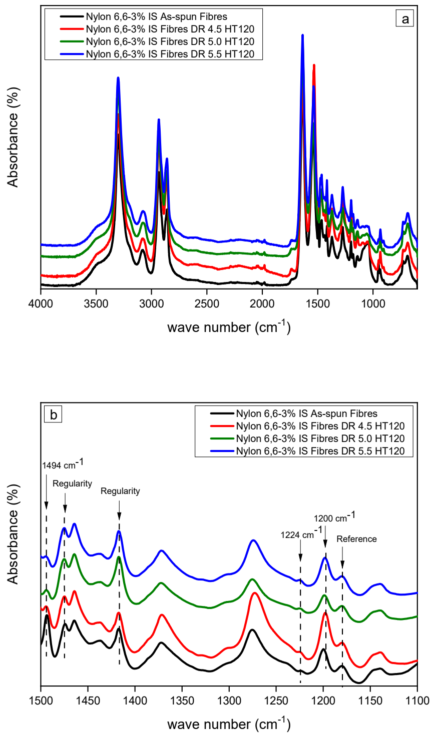

Citation: Dawelbeit, A.; Yu, M. Transient Confinement of the Quaternary Tetramethylammonium Tetrafluoroborate Salt in Nylon 6,6 Fibres: Structural Developments for High Performance Properties. *Materials* **2021**, *14*, 2938. https://doi.org/10.3390/ma14112938

Academic Editor: Jānis Andersons

Received: 24 April 2021
Accepted: 26 May 2021
Published: 10 June 2021

Publisher's Note: MDPI stays neutral with regard to jurisdictional claims in published maps and institutional affiliations.

Copyright: © 2021 by the authors. Licensee MDPI, Basel, Switzerland. This article is an open access article distributed under the terms and conditions of the Creative Commons Attribution (CC BY) license (http://creativecommons.org/licenses/by/4.0/).

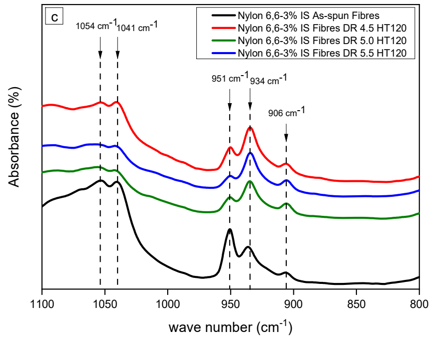

Figure S1. The FTIR spectrum of the as-spun and drawn salt-confined nylon 6,6 fibres at the drawing temperature of 120 °C at the band regions of (**a**) 4000–600 cm^{-1}, (**b**) 1500–1100 cm^{-1} and (**c**) 1100–800 cm^{-1}.

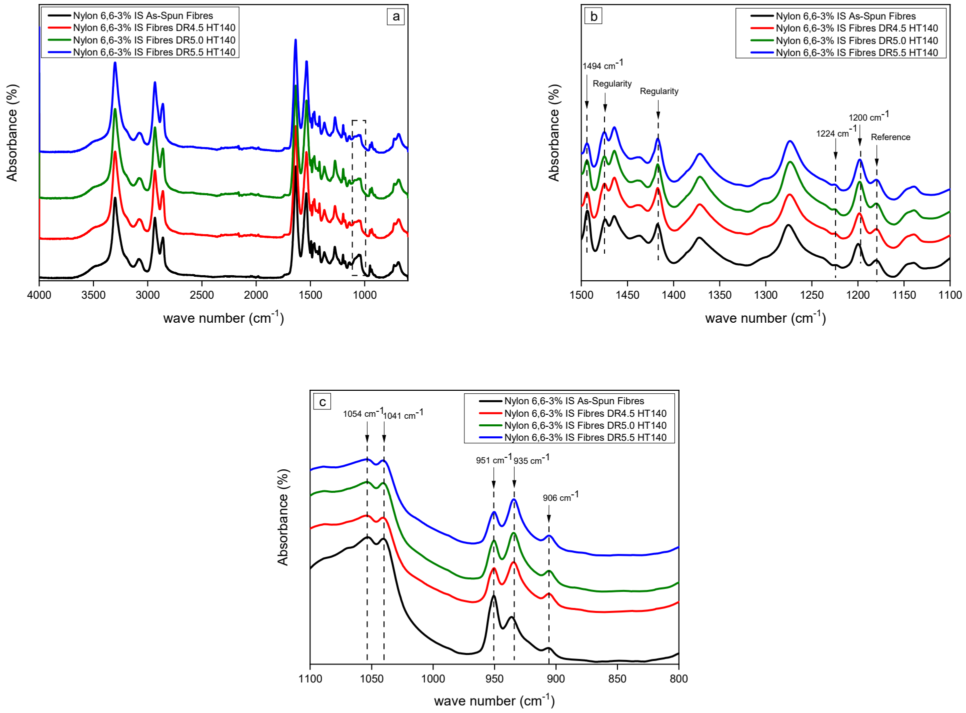

Figure S2. The FTIR spectrum of the as-spun and drawn salt-confined nylon 6,6 fibres at the drawing temperature of 140 °C at the band regions of (**a**) 4000–600 cm^{-1}, (**b**) 1500–1100 cm^{-1} and (**c**) 1100–800 cm^{-1}.

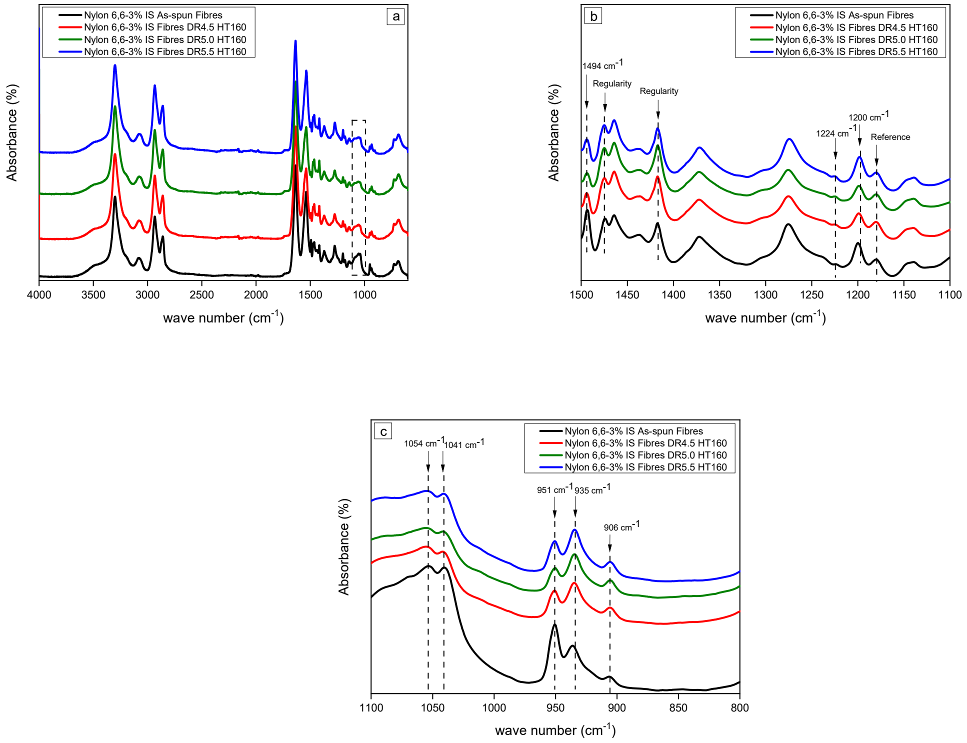

Figure S3. The FTIR spectrum of the as-spun and drawn salt-confined nylon 6,6 fibres at the drawing temperature of 160 °C at the band regions of (**a**) 4000–600 cm^{-1}, (**b**) 1500–1100 cm^{-1} and (**c**) 1100–800 cm^{-1}.

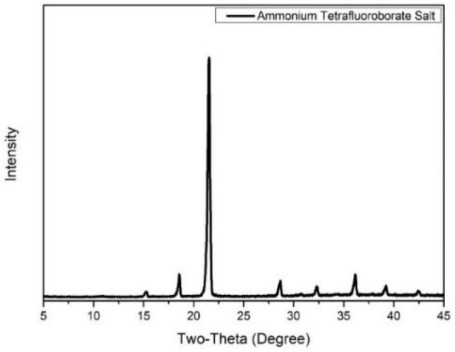

Figure S4. XRD of the TMA BF_4 salt.

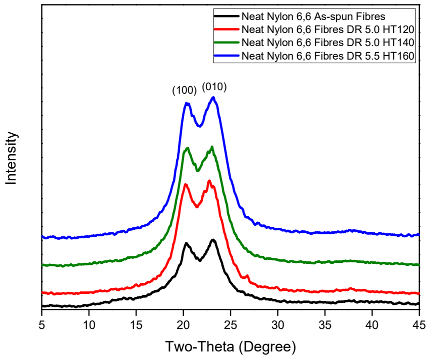

Figure S5. XRD pattern as-spun and drawn neat nylon 6,6 fibres, drawn at maximum draw ratio and at drawing temperature of 120, 140 and160 °C.

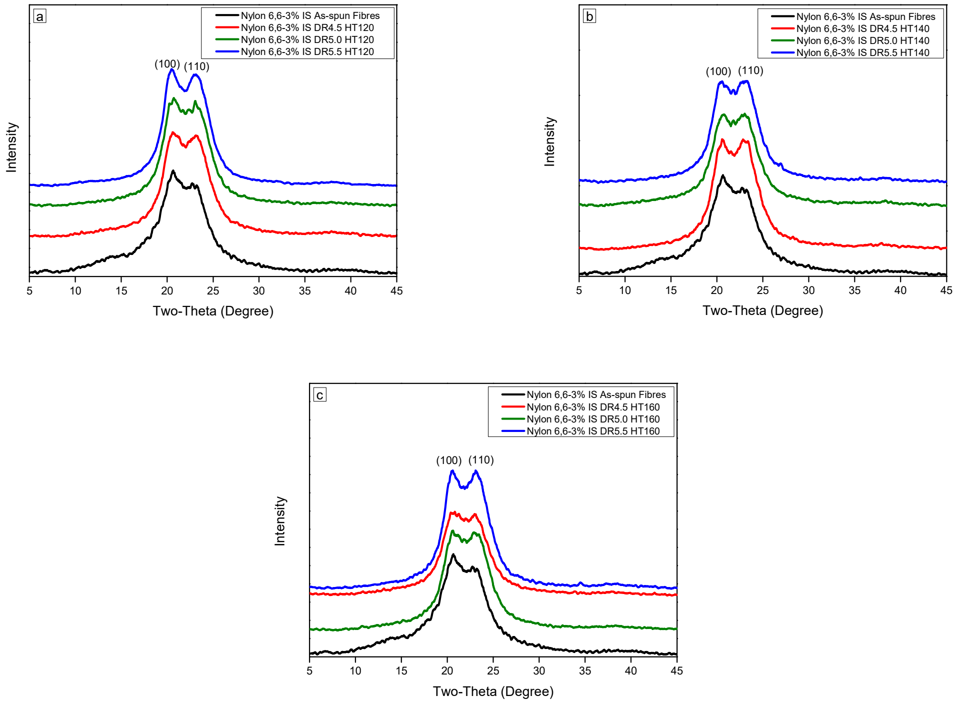

Figure S6. XRD pattern of 3% salt-confined as-spun and drawn nylon 6,6 fibres at drawing temperature of (**a**) 120, (**b**) 140 and (**c**) 160 °C.

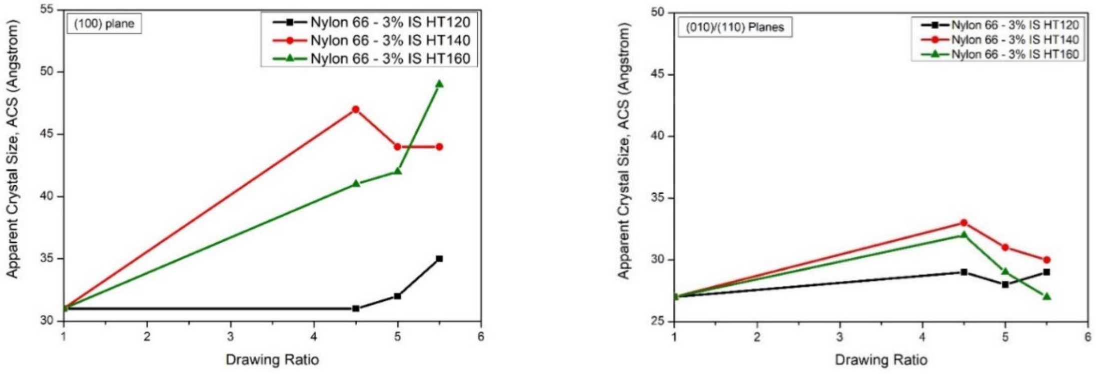

Figure S7. The ACS of the salt-confined nylon 6,6 fibres for the (100) and the (010/110) diffraction planes versus the drawing ratio at different drawing temperatures.

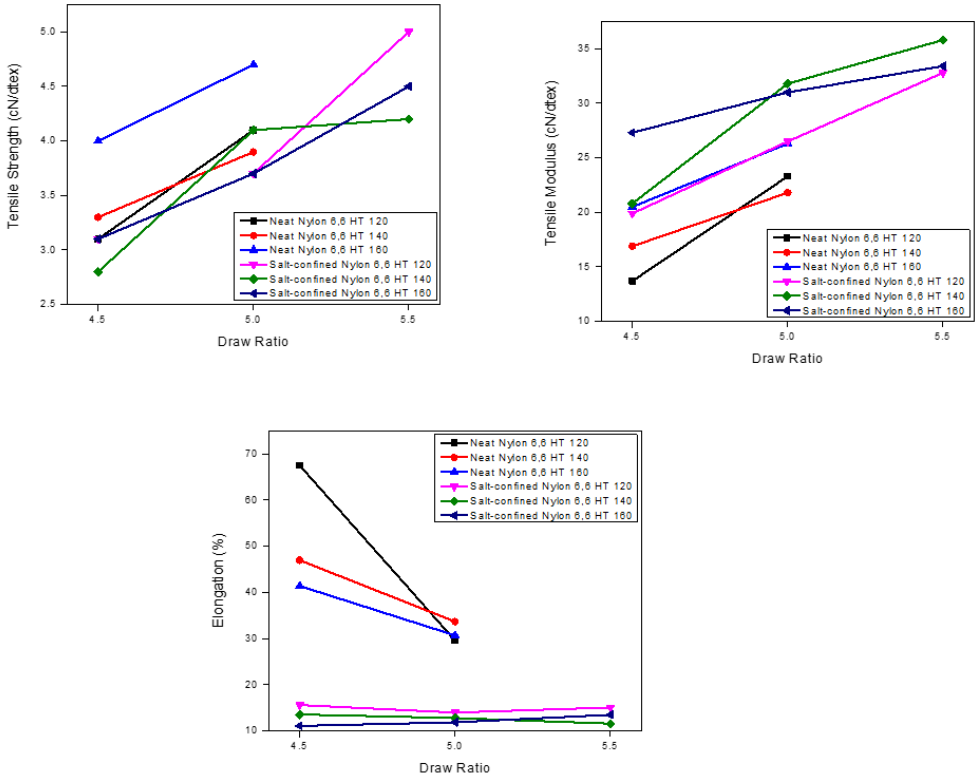

Figure S8. The tensile strengths, tensile moduli and elongation of the neat and the salt-confined nylon 6,6 fibres versus the drawing ratio at drawing temperatures of 120, 140 and 160 °C.

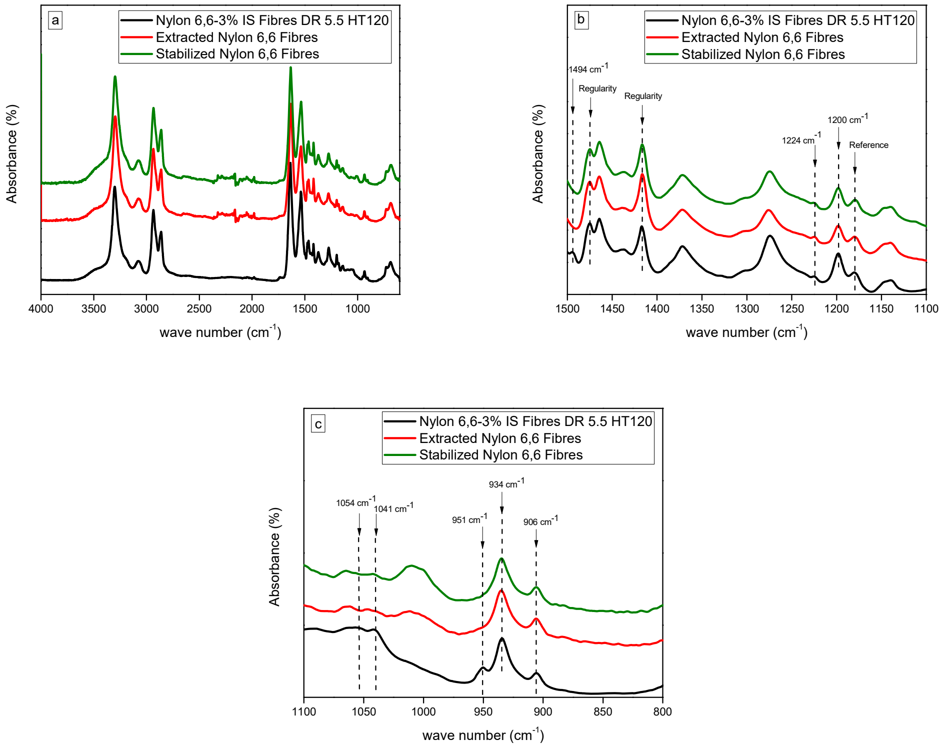

Figure S9. The FTIR spectrum of the salt-confined (drawn at, maximum, 5.5 draw ratio and at drawing temperature of 120 °C) and reverted nylon 6,6 fibres at the band regions of (**a**) 4000–600 cm^{-1}, (**b**) 1500–1100 cm^{-1} and (**c**)1100–800 cm^{-1}.

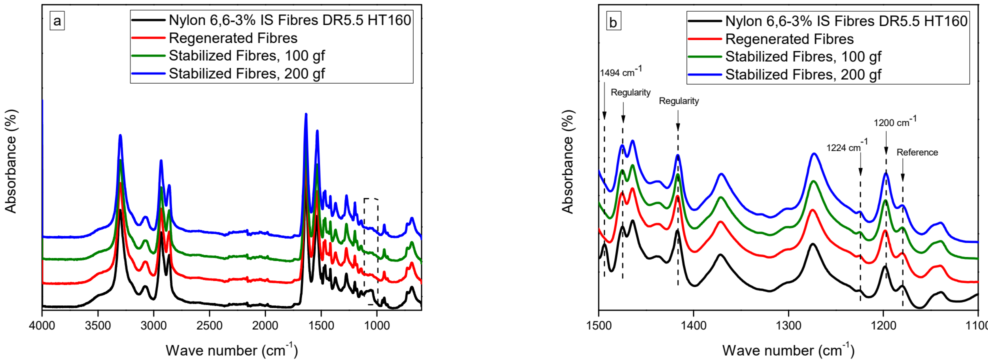

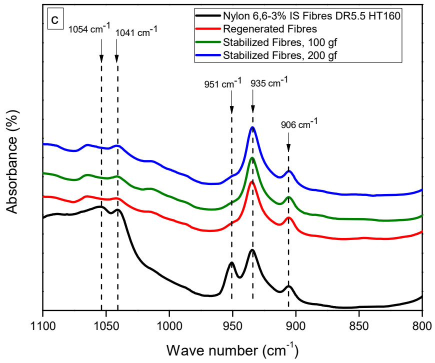

Figure S10. The FTIR spectrum of the salt-confined (drawn at, maximum, 5.5 draw ratio and at drawing temperature of 160 °C) and reverted nylon 6,6 fibres at the band regions of (**a**) 4000–600 cm^{-1}, (**b**) 1500–1100 cm^{-1} and (**c**)1100–800 cm^{-1}.

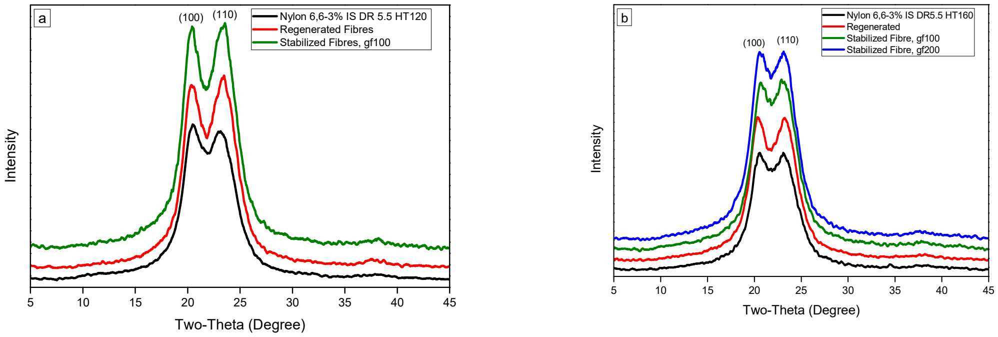

Figure S11. XRD pattern of salt-confined nylon 6,6 fibres that drawn at, maximum, 5.5 draw ratio and at drawing temperature of (**a**) 120 and (**b**) 160 °C and its reverted fibres.

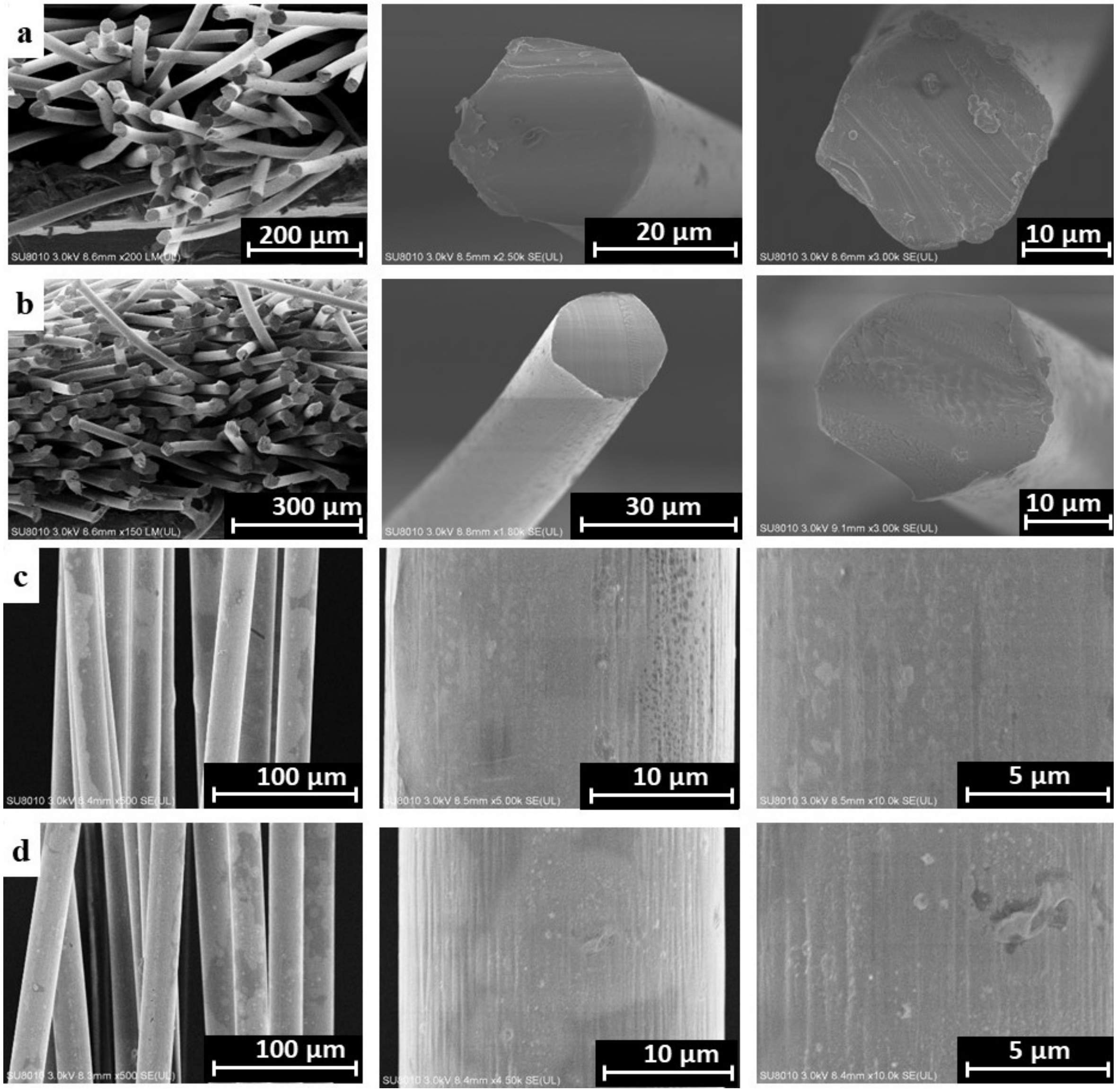
a
b
c
d
200 µm
20 µm
10 µm
300 µm
30 µm
10 µm
100 µm
10 µm
5 µm
100 µm
10 µm
5 µm

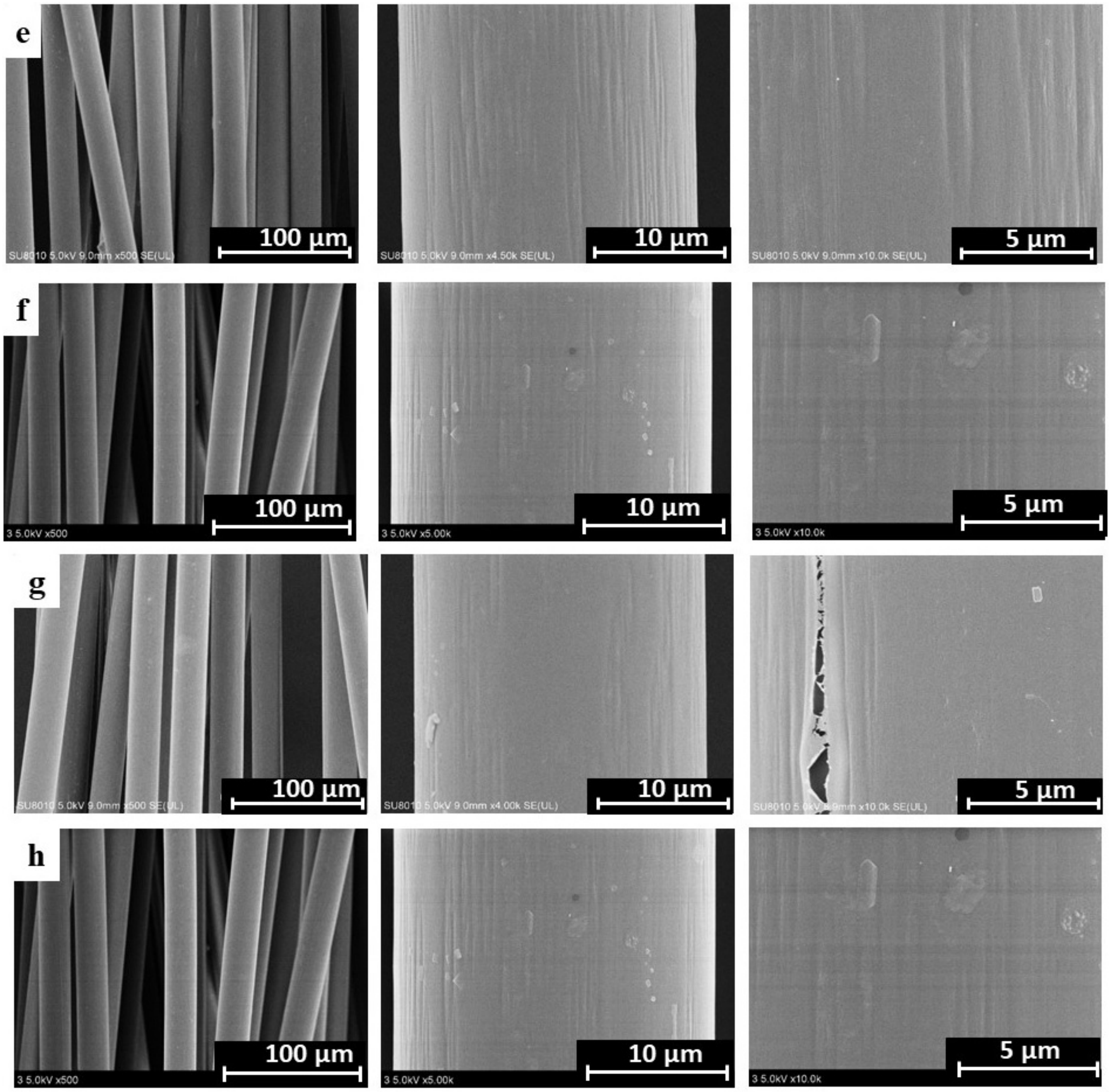

Figure S12. SEM micrographs of the reverted nylon 6,6 fibres. The morphological cross-section structures of (**a**) the regenerated and (**b**) the stabilized nylon 6,6 fibres (the regeneration have been done for the 3% salt-confined nylon 6,6 fibres drawn at drawing ratio of 5.5 and drawing temperature of 120 °C). The morphological surfaces structures of (**c**) the regenerated and (**d**) the stabilized nylon 6,6 fibres (the regeneration have been done for the 3% salt-confined nylon 6,6 fibres drawn at drawing ratio of 5.5 and drawing temperature of 120 °C). The morphological surfaces structures of (**e**) the regenerated and (**f**) the stabilized nylon 6,6 fibres (the regeneration have been done for the 3% salt-confined nylon 6,6 fibres drawn at drawing ratio of 5.5 and drawing temperature of 140 °C). The morphological surfaces structures of (**g**) the regenerated and (**h**) the stabilized nylon 6,6 fibres (the regeneration have been done for the 3% salt-confined nylon 6,6 fibres drawn at drawing ratio of 5.5 and drawing temperature of 160 °C).

2. Supplemental Tables

Table S1. Crystallographic properties of the as-spun and drawn neat nylon 6,6 fibres.

Samples	Crystallinity (%)	Interplanar space (d-spacing), Å		Crystal perfection, CPI (%)	Apparent crystal size, ACS (Å)		$cos^2\emptyset_{hkl}$	Degree of Orientation (f_c)	Orientation angle (degree)
		100	*010*		*100*	*010*			
As-spun Neat Nylon 6,6 Fibre	38.24	4.3671	3.8541	70.426	70	56	0.238	-0.144	60.84
Neat Nylon 6,6 Fibres DR 5.0 HT120	62.81	4.3959	3.8946	68.104	51	34	0.913	0.87	17.14
Neat Nylon 6,6 Fibres DR 5.0 HT140	59.35	4.3763	3.8854	66.85	51	32	0.810	0.715	25.83
Neat Nylon 6,6 Fibres DR 5.5 HT160	67.72	4.3680	3.8402	72.72	34	31	0.750	0.625	30.00

Table S2. Crystal Structural Parameters of Salt-confined and Reverted Nylon 6,6 Fibres.

Samples	Treatment Temperature (°C)	Tension (gram force)	Crystallinity (%)	Apparent crystal size, ACS (Å)		Crystal perfection, CPI (%)	$cos^2\emptyset_{hkl}$	Degree of Orientation (f_c)	Orientation angle (degree)
				100	*010*				
Salt-confined Fibres	120	DR 5.5	72.58	35	29	72.22	0.76	0.640	29.34
Regenerated Fibres	RT	-	79.31	38	30	80.08	0.728	0.592	31.46
Stabilized Fibres	190	100	82.36	39	29	79.79	0.924	0.886	16.01
Salt-confined Fibres	140	DR 5.5	58.02	44	30	72.35	0.928	0.892	15.55
Regenerated Fibres	RT	-	60.82	33	31	72.75	0.785	0.677	27.64
Stabilized Fibres	190	100	74.99	34	32	67.92	0.774	0.661	28.40
Stabilized Fibres	190	200	70.82	40	29	67.09	0.931	0.897	15.20
Salt-confined Fibres	160	DR 5.5	54.86	49	27	67.74	0.886	0.829	19.72
Regenerated Fibres	RT	-	71.98	37	30	75.43	0.902	ddd	18.25
Stabilized Fibres	190	100	71.15	36	30	65.75	0.861	0.791	21.92
Stabilized Fibres	190	200	68.37	36	30	69.90	0.860	0.790	21.97
